# Multiple excitatory actions of orexins upon thalamo-cortical neurons in dorsal lateral geniculate nucleus - implications for vision modulation by arousal

**DOI:** 10.1038/s41598-017-08202-8

**Published:** 2017-08-09

**Authors:** Lukasz Chrobok, Katarzyna Palus-Chramiec, Anna Chrzanowska, Mariusz Kepczynski, Marian Henryk Lewandowski

**Affiliations:** 1 Department of Neurophysiology and Chronobiology, Institute of Zoology and Biomedical Research, Jagiellonian University in Krakow, Gronostajowa 9 Street, 30-387 Krakow, Poland; 2 Faculty of Chemistry, Jagiellonian University in Krakow, Ingardena 3 Street, 30-060 Krakow, Poland

## Abstract

The orexinergic system of the lateral hypothalamus plays a crucial role in maintaining wakefulness and mediating arousal in a circadian time-dependent manner. Due to the extensive connections of orexinergic neurons, both orexins (OXA and OXB) exert mainly excitatory effects upon remote brain areas, including the thalamus. The dorsal lateral geniculate nucleus (DLG) is a relay thalamic centre for the visual system. Its thalamo-cortical (TC) neurons convey photic information from the retina to the primary visual cortex. The present study shows that orexins are powerful modulators of neuronal activity in the DLG. OXA directly depolarised the majority of neurons tested, acting predominately on postsynaptic OX_2_ receptors. Moreover, OXA was found to increase excitability and enhance neuronal responses to both glutamate and γ-aminobutyric acid (GABA). Mechanistic studies showed the involvement of voltage-gated calcium currents and GIRK channels in the observed depolarisations. Immunohistochemical staining showed sparse orexinergic innervation of the DLG during the light phase, with increased density at night. We hypothesise that the depolarising effects of orexins upon DLG neurons may facilitate signal transmission through the visual thalamo-cortical pathway during behavioural arousal. Thus, the action of orexin on DLG TC neurons may underlie the circadian/behavioural modulation of vision.

## Introduction

The orexins/hypocretins (orexin A/hypocretin 1 and orexin B/hypocretin 2) are two neuropeptides synthesised from a common precursor in a group of neurons localised in the lateral hypothalamus and perifornical area^1–3^ that extensively innervate many nuclei in the brain^4^. Orexins bind to two G protein-coupled receptors: the orexin-1 (OX_1_) and orexin-2 (OX_2_) receptors; activation results in membrane depolarisation and an increase in excitability^2, 5, 6^. Several ionic mechanisms are known to underlie the excitatory effects of orexins, including calcium influx^7–11^ and the closure of potassium channels^12–16^. Growing evidence suggests a major role of the orexinergic system in maintaining wakefulness^17–19^ and arousal^20, 21^; the depletion of orexinergic neurons or alterations in the OX_2_ receptor gene cause narcolepsy^22^. Moreover, the activity of orexinergic neurons is dependent on circadian time and light^23, 24^.

The dorsal lateral geniculate nucleus (DLG) is a primary visual thalamic structure that relays information from the retina to the primary visual cortex. Besides photic input, thalamo-cortical (TC) neurons are depolarised by neurotransmitters such as acetylcholine or noradrenaline, which promotes sensory transmission during arousal^25, 26, 27^. These classical neurotransmitters are known to modulate signal transmission from the thalamus to the cortex across the sleep-wake cycle^28, 29^, and a similar role of the orexinergic system at the level of the thalamus has been suggested^12, 30^. Interestingly, the circadian modulation of visual sensitivity has been noted in many species of invertebrates, as well as in rats and humans^31, 32^.

It has been proposed that orexins do not influence the activity of DLG neurons^12, 33^. Nevertheless, the linkage between orexinergic and visual systems has been explored. Orexins exclusively activate neurons in layer 6b of the visual cortex, which reciprocally project to the thalamus^26, 34, 35^ and are expressed and active in the retina^36, 37^. Recently, it has been also shown that orexins affect brain structures involved in the processing of retinal information such as the suprachiasmatic nucleus of the hypothalamus^38–40^, the intergeniculate leaflet^41, 42^ or the ventral lateral geniculate nucleus of the thalamus^43^.

The aim of this study was to determine if orexin A (OXA) and orexin B (OXB) have modulatory effects upon DLG TC neuronal activity. First, we characterised the excitatory responses to orexins and determined the receptor involved in OXA-evoked depolarisation. Subsequently, we focused on explaining the discrepancies between our results and those of others^12, 33^. In the next step, we investigated the changes in excitability and responses to basic neurotransmitters evoked by OXA and unravelled the ionic mechanism underlying the observed depolarisation. Finally, we performed an immunohistochemical study on the circadian changes in the density of orexin-immunoreactive (orexin-ir) fibres in the area of the DLG. To the best of our knowledge, the present study shows for the first time the excitatory effects of orexins on the primary visual thalamus.

## Results

**Figure 1:**
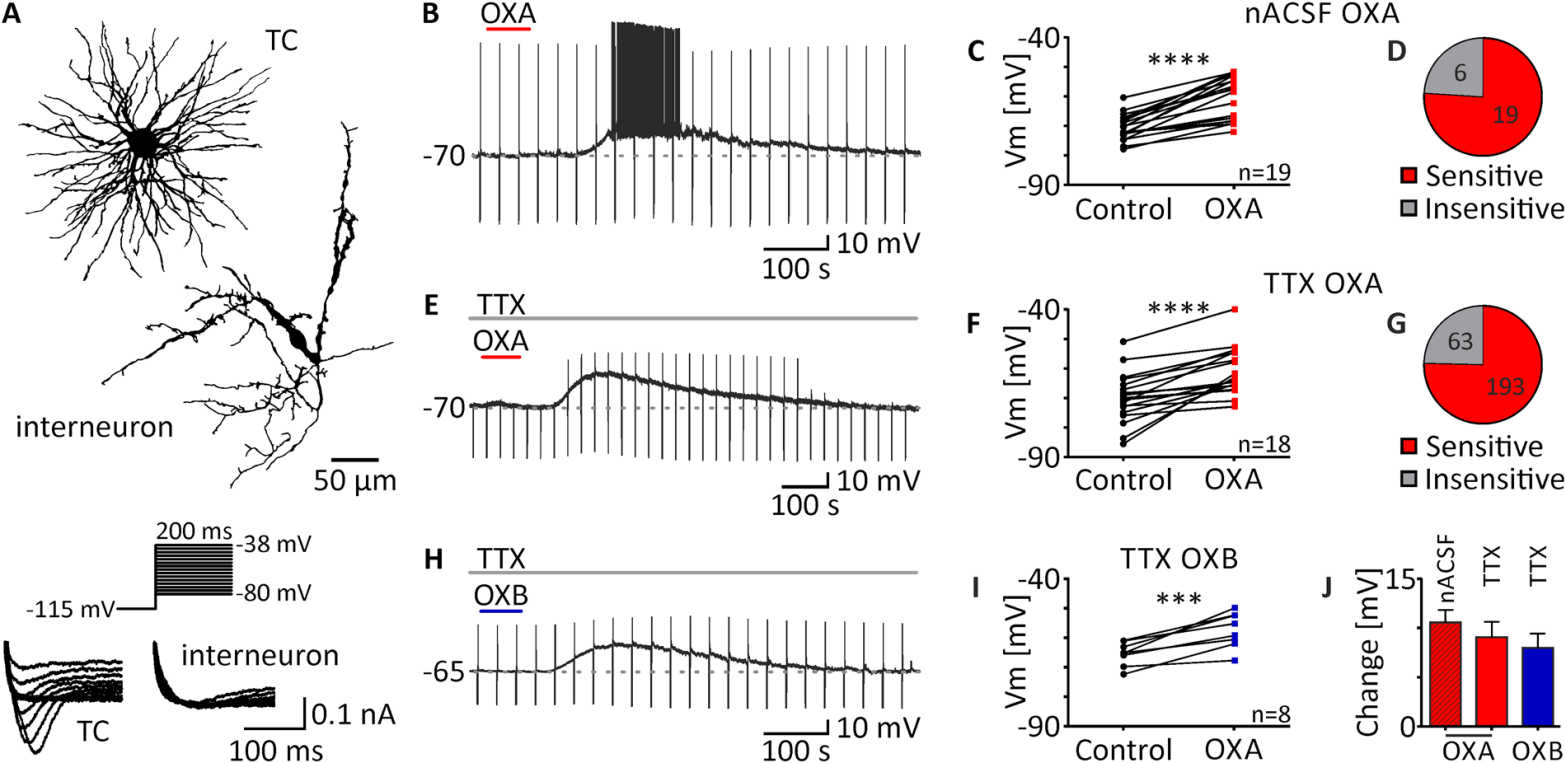
Orexins excite thalamo-cortical neurons in the DLG. (**A**) Typical morphology of a thalamo-cortical neuron (TC) and an interneuron. Current responses to the voltage step protocol also differed between cell types (*below*). (**B**–**D**) OXA application (200 nM, red bar) in normal ACSF (nACSF) caused robust excitation in the majority of neurons tested (n = 19/25). (**E**,**F**) Depolarisations evoked by OXA remained in the presence of TTX (0.5 μM, grey bar) in the ACSF. (**G**) Statistics based on the sensitivity of all neurons tested in this study in the presence of TTX in all experimental protocols show that OXA activated about three quarters of DLG neurons examined (n = 193/256). (**H,I**) Similar direct depolarisations were recorded after the application of OXB (200 nM, blue bar) in the presence of TTX. (**J**) No significant differences were noted between OXA-evoked effects (in red) in nACSF (striped) and TTX (plain; no network-dependent effects) nor between OXA- and OXB-evoked depolarisations (in blue). ***p < 0.001, ****p < 0.0001 denotes significance (paired *t* test or Wilcoxon test). In the bar graphs, data are expressed as mean ± SEM. The number of responsive neurons is shown as “n”. Downward deflections in the raw current clamp traces represent responses to rectangular current pulses (1 s, 80 pA).

Two distinct subpopulations of neurons can be found in the DLG: excitatory thalamo-cortical (TC) neurons that project an axon to the primary visual cortex and GABAergic interneurons, easily identified by their characteristic morphology and electrophysiology^44, 45^ (Fig. 1A). In our patch-clamp study, we focused on TC neurons (large multipolar cells with the T-type calcium conductance of a high amplitude) to examine their possible sensitivity to orexins. All in all, neurons derived from 121 rats were recorded and each experimental protocol was performed on the brain slices from the minimum number of four rats. Additional 24 rats were used for circadian immunohistochemical studies.

### TC neurons in DLG are directly excited by orexins

First, we applied OXA (200 nM) to 25 TC DLG neurons in normal ACSF (nACSF; without any other pharmacological agents). All of the recorded neurons were silent, generating only a rebound burst of sodium/calcium spikes after recovery from pulse-evoked hyperpolarisation. In 19 neurons tested, OXA evoked robust depolarisation (from −70.66 ± 1.0 mV to −60.11 ± 1.7 mV; *p* < 0.0001, paired *t* test, n = 19; Fig. 1B,C), while the remaining six cells were insensitive to peptide application (Fig. 1D). Excitation was accompanied by the appearance of action potentials in eight sensitive neurons (firing frequency: 1.67 ± 0.57 Hz; *p* = 0.0221, paired *t* test, n = 8). A second application of OXA in the presence of TTX (0.5 μM) and blockers of fast synaptic transmission in ACSF (CNQX, 10 μM; AP-5, 40 μM; Bic, 20 μM) was performed on eight sensitive neurons to check if the observed effects were direct and postsynaptic. The observed depolarisation persisted in all neurons tested (from −70.21 ± 1.9 mV to −65.72 ± 2.5 mV; *p* = 0.0097, paired *t* test, n = 8). In the next step, a group of 30 DLG TC neurons was tested by a single application of OXA in the presence of TTX in ACSF; 12 neurons remained insensitive, whilst 18 were depolarised (from −69.88 ± 2.0 mV to −60.81 ± 1.9 mV; *p* < 0.0001, paired *t* test, n = 18; Fig. 1E,F). During all protocols presented in this article, 256 neurons were tested in the presence of TTX, of which 193 (75.39%) were depolarised and 63 (24.61%) were insensitive to OXA application (Fig. 1G). Under the same conditions, OXB (200 nM) was applied to eight DLG TC neurons and caused depolarisation in all cells tested (from −65.56 ± 1.4 mV to −57.59 ± 2.1 mV, *p* = 0.0009, paired *t* test, n = 8; Fig. 1H,I). No significant changes were noted between the application of OXA in nACSF and in ACSF + TTX (mean change in nACSF: 10.55 ± 1.3 mV, n = 19; in TTX: 9.07 ± 1.5 mV, n = 18, *p* = 0.4624, unpaired *t* test; Fig. 1J) nor between responses to OXA and OXB in the presence of TTX (OXA: 9.07 ± 1.5 mV, n = 18; OXB: 7.98 ± 1.5 mV, n = 8, *p* = 0.6671 unpaired *t* test; Fig. 1J).

### Orexin-evoked depolarisation of TC DLG neurons is independent of the rat strain, anaesthesia and intrapipette chloride concentration

**Figure 2:**
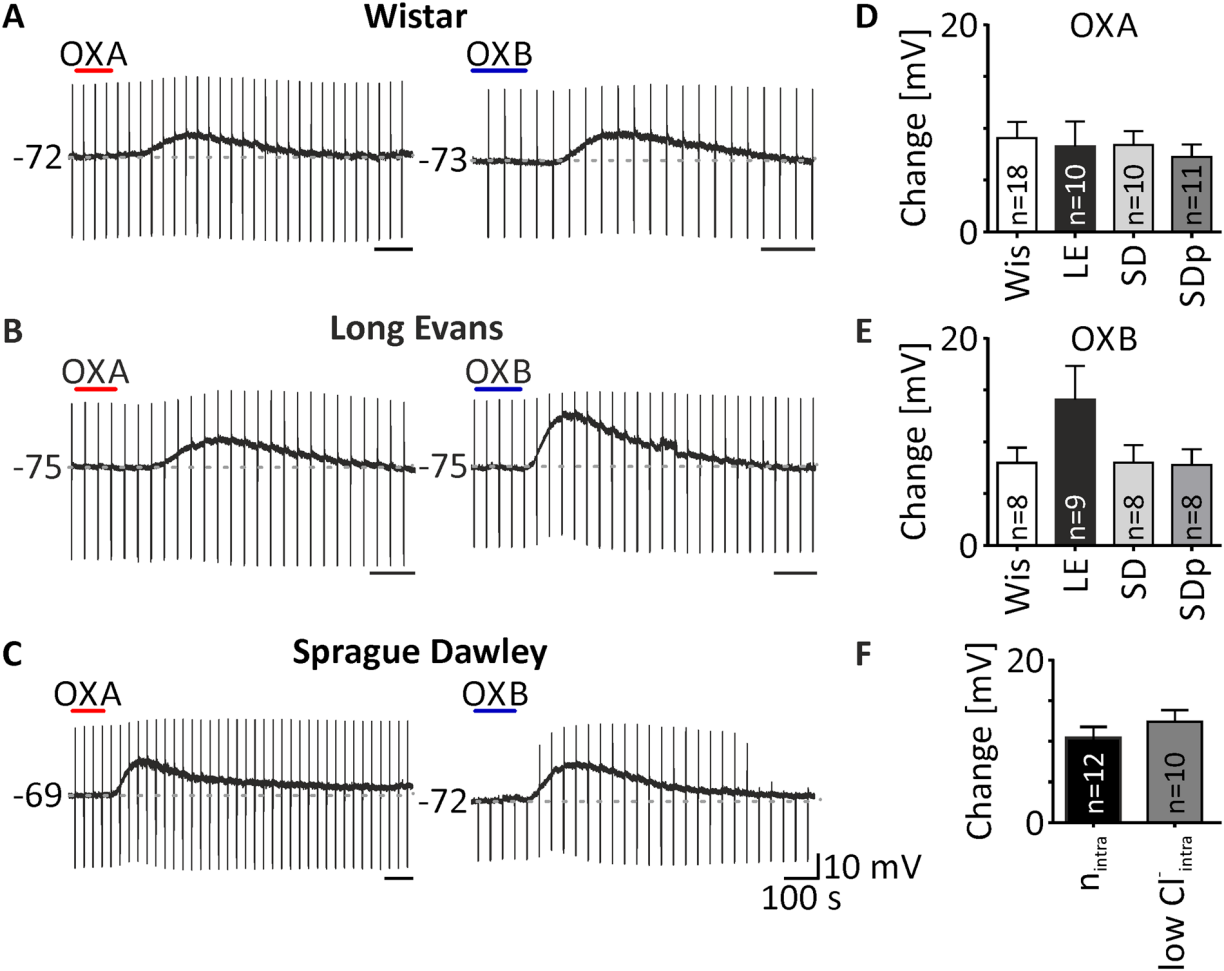
Depolarisations evoked by orexins in the DLG are independent on the rat strain, type of anaesthesia and chloride reversal potential (R_Cl_^−^). (**A–C**) Example recordings showing OXA- (200 nM, red bar) and OXB-evoked (200 nM, blue bar) depolarisations of DLG TC neurons in Wistar (Wis, **A**), Long Evans (LE, **B**) and Sprague Dawley rats (SD, **C**). No significant differences in amplitude of OXA- (**D**) and OXB-evoked depolarisations (**E**) were noted between rat strains and between the types of anaesthesia used, tested in Sprague Dawley rats (SDp - rats treated with pentobarbital instead of isoflurane). (**F**) A change in the chloride ion concentration in the intrapipette solution did not change either the sensitivity or the depolarisation amplitude of the OXA effects in nACSF (n_intra_ - normal intrapipette, R_Cl_^−^ of −43 mV; lowCl^−^_intra_ - low chloride intrapipette, R_Cl_^−^ of −70 mV). In the bar graphs, data are expressed as mean ± SEM. The number of responsive neurons is shown as “n”. Downward deflections in the raw current clamp traces represent responses to rectangular current pulses (1 s, 80 pA).

The next step was devoted to excluding possible differences between experimental designs that would explain the discrepancies between our results (DLG sensitivity to orexins) and those of others^12, 33^. First, we checked if the sensitivity to both orexins was strain-specific; all of these experiments were performed in the presence of TTX (0.5 μM) in the ACSF. We applied OXA (200 nM) to 14 DLG TC neurons from pigmented Long Evans rats and also recorded robust depolarisations in 10 cases (from −72.94 ± 2.3 mV to −64.67 ± 4.1 mV, *p* = 0.0074, paired *t* test, n = 10; Fig. 2B,D), while four neurons were insensitive to this treatment. Similarly, we applied OXB (200 nM) to 11 DLG TC neurons, of which nine were excited by the peptide (from −74.85 ± 1.2 mV to −60.77 ± 3.8 mV, *p* = 0.0026, n = 9; Fig. 2B,E). We then explored the sensitivity to orexins in Sprague Dawley rats, in which DLG TC neurons have been suggested to be unresponsive to both OXA and OXB^12, 33^. The application of OXA to 18 DLG TC neurons resulted in ten depolarisations (from −71.50 ± 1.5 mV to −63.10 ± 2.5 mV, *p* = 0.0001, paired *t* test, n = 10; Fig. 2C,D), whereas eight neurons remained insensitive to this treatment. OXB application to 10 cells caused eight responses (from −71.14 ± 1.6 mV to −63.12 ± 2.2 mV, *p* = 0.0019, paired *t* test, n = 8; Fig. 2C,E), but in two cases did not evoke depolarisation. In addition, we performed a set of experiments, also on Sprague Dawley rats, where isoflurane anaesthesia was changed to sodium pentobarbital i.p. injection, as in the study by Govindaiah and Cox (2006). In this case, OXA was applied to 14 DLG TC neurons and, again, caused excitation in the majority (n = 11/14) of tested cells (from −70.96 ± 1.0 mV to −63.72 ± 1.5 mV, *p* = 0.0001, paired *t* test, n = 11; Fig. 2D). The application of OXB evoked depolarisations in all eight neurons examined (from −70.15 ± 1.4 mV to −62.39 ± 1.9 mV, *p* = 0.0014, paired *t* test, n = 8; Fig. 2E).

Then, we shifted our attention to the intrapipette chloride concentration, which was another variable differentiating our design from those of others^12, 33^. We tested responses to OXA (200 nM) in DLG TC neurons from Wistar rats with the use of a low Cl^−^ intrapipette solution. All of the tested neurons were silent. OXA was applied to 10 neurons in nACSF, all of which were excited (from −69.70 ± 1.6 mV to −57.29 ± 1.9 mV, *p* < 0.0001, paired *t* test, n = 10). Five neurons generated action potentials during the OXA-evoked depolarisation (firing frequency: 1.56 ± 0.4 Hz). In the case of seven neurons, OXA was applied again in the presence of TTX. All of them were directly excited by the peptide (from −74.71 ± 1.6 mV to −68.47 ± 1.8 mV, *p* = 0.0003, paired *t* test, n = 7). Having collected these data, we then compared the mean depolarisation evoked by OXA in nACSF recorded with our normal intrapipette solution (n_intra_) with that recorded with the low Cl^−^ concentration (lowCl^−^_intra_). No significant differences were noted (n_intra_: 10.55 ± 1.3 mV, n = 19; lowCl^−^_intra_: 12.41 ± 1.4 mV, n = 10, *p* = 0.3683, unpaired *t* test; Fig. 2F).

### Both orexin receptors are involved in the OXA effect in the DLG, with the predominance of the OX_2_ receptor

**Figure 3:**
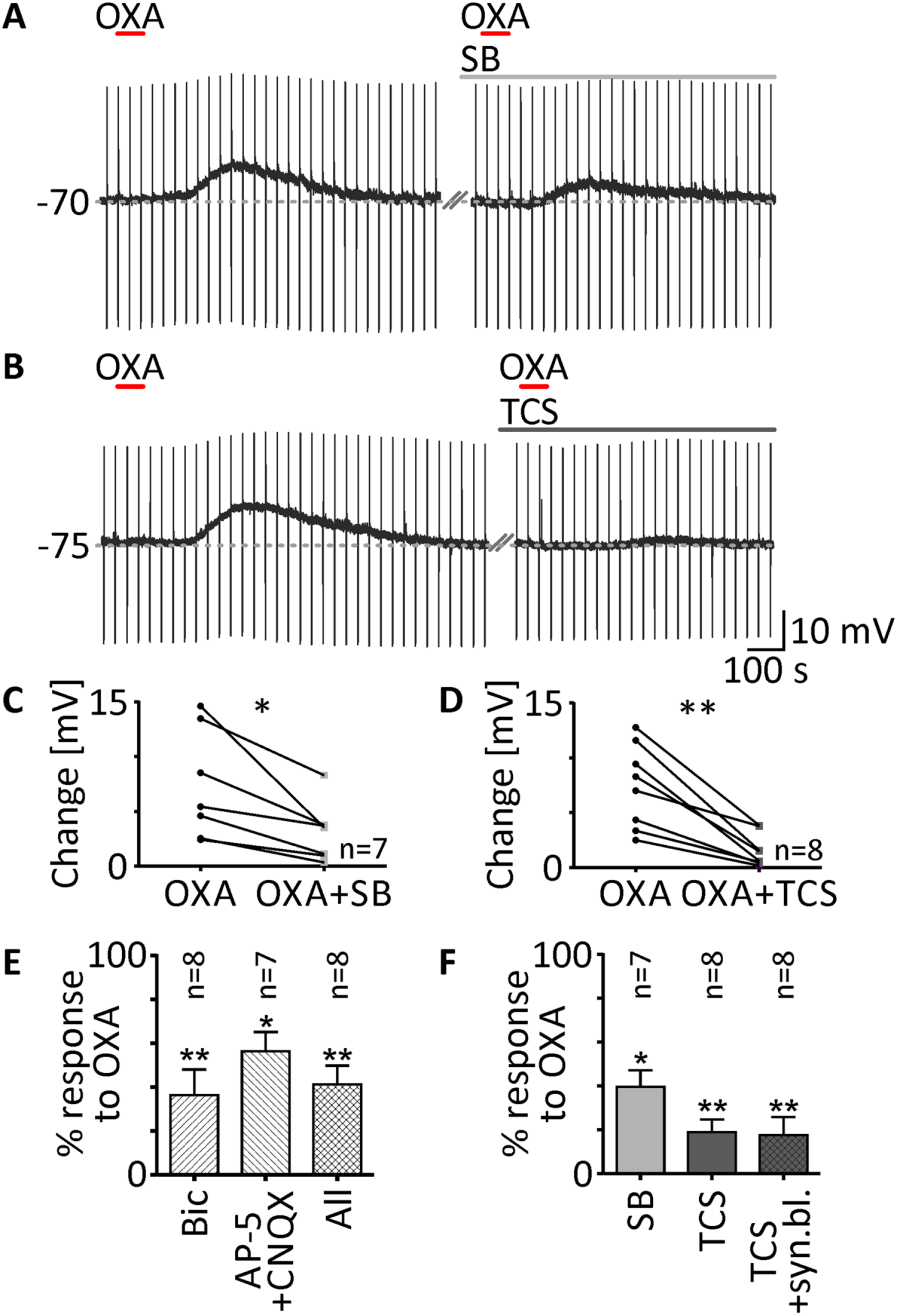
OXA evokes depolarisation of DLG TC neurons predominately via the postsynaptic OX_2_ receptor. (**A**,**B**) Raw recording traces showing OXA-evoked depolarisations (200 nM, red bar) in TTX (0.5 μM) and second responses in the presence of the selective OX_1_ receptor antagonist SB 334867 (SB, 10 μM, light grey bar; (**A**) or TCS OX2 029 (TCS, 10 μM, dark grey bar; (**B**) - a selective blocker of OX_2_ receptors. SB was able to partially inhibit depolarisations evoked by OXA (**C**,**F**), although TCS was much more potent (**D**,**F**). (**E**) Effects of OXA observed in the DLG were not exclusively dependent on postsynaptic receptors as the application of this peptide with a blocker of fast synaptic transmission such as bicuculline (Bic), AP-5 and CNQX showed reduced effects. Application of OXA in the presence of synaptic blockers and TCS together indicated the postsynaptic localisation of the OX_2_ receptor (**F**). *p < 0.05, **p < 0.01 denotes significance (paired *t* test or Wilcoxon test). In the bar graphs, data are expressed as mean ± SEM. The number of examined neurons is shown as “n”. Downward deflections in the raw current clamp traces represent responses to rectangular current pulses (1 s, 80 pA).

Subsequently, we investigated the possible involvement of both orexin receptors in the observed depolarisations after OXA (200 nM) application to DLG TC neurons. Each time, OXA was applied twice. The first application was performed in the presence of TTX (0.5 μM) only, whereas the second one was done with the addition of a specific blocker. First, we studied the effects of SB 334867 (10 μM; the selective OX_1_receptor antagonist) on seven responsive DLG TC neurons. The response to OXA in the presence of this antagonist was reduced to 39.57 ± 7.6% (depolarisation under control conditions: 7.38 ± 1.9 mV; SB: 3.11 ± 1.0 mV, *p* = 0.0147, paired *t* test, n = 7; Fig. 3A,C,F). Next, in the case of eight neurons, second application of OXA was performed after the blockage of OX_2_ receptors with TCS OX2 029 (10 μM). This antagonist reduced the response to OXA to 19.08 ± 5.7% (control: 7.41 ± 1.3 mV; TCS: 1.54 ± 0.5 mV, *p* = 0.0011, paired *t* test, n = 8; Fig. 3B,D,F).

In order to check if orexin receptors are localised exclusively on the postsynaptic side, we performed a set of experiments with various synaptic blockers, using the same protocol described above. First, in the case of eight neurons, we blocked fast GABAergic transmission with bicuculline (20 μM). This manipulation dampened the second response to OXA to 36.25 ± 11.8% (control: 13.39 ± 1.7 mV; Bic: 4.21 ± 1.2 mV, *p* = 0.0011, n = 8; Fig. 3E). Similarly, having blocked fast glutamatergic transmission with a mixture of AP-5 (40 μM) and CNQX (10 μM) in seven neurons, we observed a reduction in the second response to OXA to 56.29 ± 8.8% (control: 9.31 ± 2.8 mV; AP-5 + CNQX: 5.01 ± 1.5 mV, *p* = 0.0356, paired *t* test, n = 7; Fig. 3E). In the next group of eight neurons, we applied OXA in a cocktail of ionotropic GABA and glutamate receptor antagonists. Under these conditions, the second OXA-evoked depolarisation was reduced to 41.25 ± 8.6% (control: 12.31 ± 2.1 mV; Bic + AP-5 + CNQX: 4.50 ± 1.3 mV, *p* = 0.0078, Wilcoxon test, n = 8; Fig. 3E).

To answer the final question on whether the predominant OX_2_ receptors are present on the postsynaptic side of DLG TC neurons, we applied OXA in a cocktail of synaptic transmission blockers and TCS OX2 029 together. Under these conditions, the response of eight neurons to OXA was reduced to 17.61 ± 8.3% (control: 9.87 ± 1.5 mV; Bic + AP-5 + CNQX + TCS: 1.44 ± 0.5 mV, *p* = 0.0010, paired *t* test, n = 8; Fig. 3F).

### OXA increases DLG TC neuron excitability and enhances responses to basic neurotransmitters

**Figure 4:**
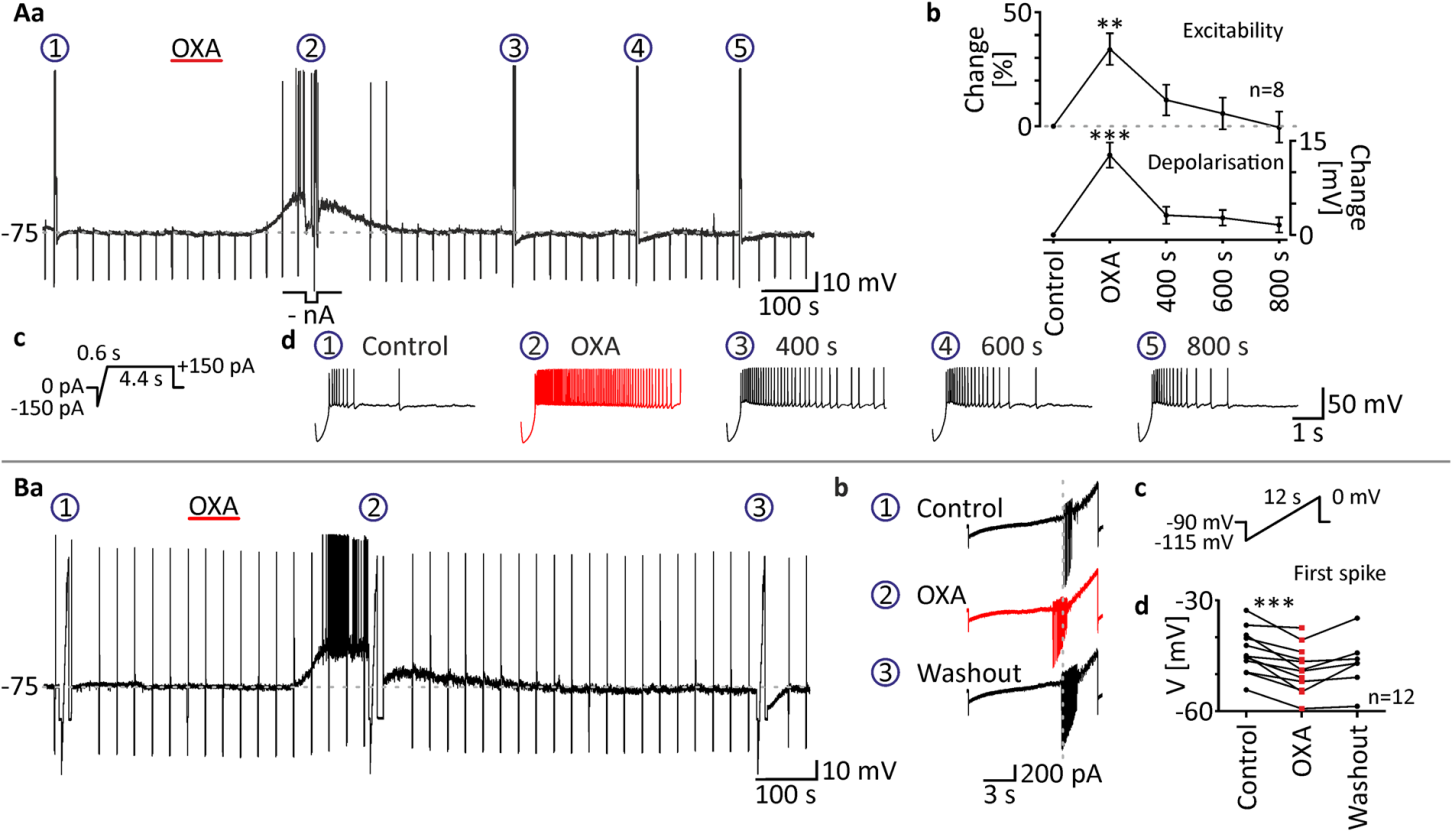
OXA increases the excitability of DLG TC neurons. (**Aa**) To examine changes in the excitability evoked by OXA (200 nM, red bar) application, trapezoidal current tests (**Ac**) were performed before drug application (circled 1), during maximal depolarisation (circled 2) and at three time points during washout (400 s, 600 s and 800 s after OXA-evoked excitation; circled 3, 4 and 5, respectively). A negative current was applied to adjust the membrane potential to control values during the effect (-nA). (**Ab**) OXA was found to evoke transient depolarisation accompanied by sustained enhancement of excitability, slowly returning to control values, expressed as an increased firing rate at the top of the current test. Enlarged responses to the current test are shown in (**Ad**). Please note the increased number of action potentials during the OXA effect (in red). (**Ba**). To check for possible changes in the sodium current threshold evoked by OXA (200 nM, red bar) application, slow voltage ramps (**Bc**) were performed before drug administration (circled 1), during the maximal effect (circled 2) and during washout (circled 3). Enlarged current responses are shown in (**Bb)**. Downward deflections represent sodium spikes. Note that during the response to OXA (in red), the threshold of sodium spikes was shifted towards more hyperpolarised values. (**Bb)**. In all neurons tested, OXA shifted the potential of first spike generation. *p < 0.05, **p < 0.01, ***p < 0.001 denotes significance (Dunn’s multiple comparison test or paired *t* test). The number of examined neurons is shown as “n”. Downward deflections in the raw current clamp traces represent responses to rectangular current pulses (1 s, 80 pA).

To address the possibility that OXA (200 nM) may change the excitability of DLG TC neurons, we conducted a set of experiments in nACSF where the trapezoidal current test (Fig. 4Ac) was performed before, during and after OXA-evoked excitation. In order to characterise the dynamics of excitability changes, the current test was repeated three times under washout conditions, 400, 600 and 800 seconds after the maximal effect of OXA (Fig. 4Aa). Action potentials fired at the top of each test were counted and this parameter was collated with membrane potential changes at each time point (Fig. 4Ad). Thus, in the eight neurons tested, OXA evoked transient membrane depolarisation (12.73 ± 2.0 mV, *p* = 0.0020, Dunn’s test, n = 8; Fig. 4Ab), accompanied by significantly increased excitability (*p* = 0.0015, Dunn’s test, n = 8; Fig. 4Ab), then slowly returned to baseline values at the three washout time points (data presented as % change from control, i.e. OXA: 33.88 ± 6.9%; 400 s: 11.50 ± 6.7%; 600 s: 5.63 ± 6.9%; 800 s: −0.50 ± 7.0%, *p* < 0.0001, Friedman test, n = 8; Fig. 4Ab).

To further examine whether OXA (200 nM) changes the threshold of action potential generation (manifested in voltage clamp as sodium inward currents), we tested 12 DLG TC neurons with the use of slow voltage ramps (Fig. 4Bc) in nACSF. These voltage tests were performed under control conditions, during maximal OXA-evoked depolarisation and (in the case of six neurons out of 12) during washout. As a result of this experiment, we found that OXA shifted the threshold of transient sodium currents towards more hyperpolarised values (control: −43.88 ± 1.7 mV; OXA: −48.68 ± 1.8 mV, *p* = 0.0002, paired *t* test, n = 12; washout: −46.93 ± 2.7 mV, n = 6; Fig. 4Bb,d).

**Figure 5:**
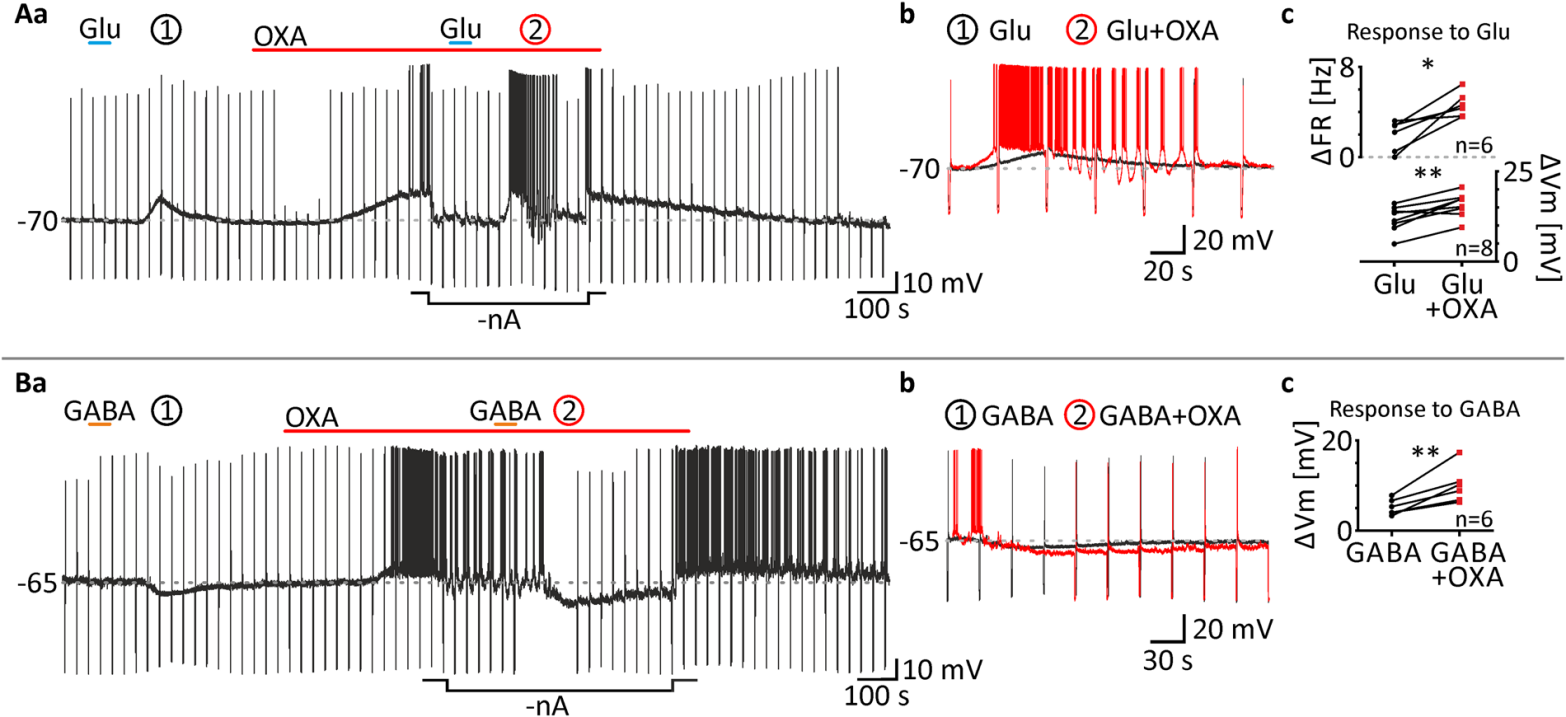
OXA enhances responses to glutamate and GABA of DLG TC neurons. (**Aa)** In order to examine if orexins modulate the glutamate-evoked activation of DLG TC neurons, glutamate (Glu, 200 μM, blue bars) was applied twice. First, the excitatory response to Glu (circled 1) was recorded under control conditions. Next, tonic application of OXA (100 nM, red bar) was performed. During the plateau of the maximal response to OXA, membrane potential was manually adjusted to control values (-nA) and Glu was applied again. (**Ab**) As shown in the enlarged traces, Glu evoked much stronger excitation in the presence of OXA (in red). (**Ac**) Tonic application of OXA increased Glu-evoked depolarisations (ΔVm) in all neurons tested. In six out of eight cells tested, OXA caused or increased neuronal firing (ΔFR) during Glu-evoked responses. (**Ba**) Corresponding experiments were carried out with GABA (100 μM, orange bars). (**Bb**) As shown at the enlarged traces, GABA was able to evoke stronger inhibition of DLG TC neurons in the presence of OXA (100 nM, in red). (**Bc**) In all neurons tested, recorded GABA-evoked hyperpolarisations (ΔVm) were exacerbated by OXA treatment. *p < 0.05, **p < 0.01, ***p < 0.001 denotes significance (paired *t* test). The number of examined neurons is shown as “n”. Downward deflections in the raw current clamp traces represent responses to rectangular current pulses (1 s, 80 pA).

Further, we looked for the possible modulation of glutamate- and GABA-evoked responses by OXA. First, we applied glutamate (200 μM) to eight DLG TC neurons in nACSF, which evoked transient membrane depolarisation. Then, OXA (100 nM) was applied tonically and during the plateau of the maximal response, and a negative current was applied to adjust the membrane potential to control values. Under these conditions, glutamate was applied again, evoking significantly stronger depolarisation (response to Glu under control conditions: 11.79 ± 1.3 mV; Glu in OXA: 15.69 ± 1.2 mV, *p* = 0.0047, paired *t* test, n = 8; Fig. 5Aa–c). In the case of six neurons tested, action potentials were generated during the first and/or second response to glutamate. The glutamate-evoked firing rate was also enhanced in the presence of OXA (control: 1.93 ± 0.6 Hz; in OXA: 4.66 ± 0.4 Hz, *p* = 0.0109, paired *t* test, n = 6; Fig. 5Ac). Similar experiments were performed with GABA (100 μM). GABA alone evoked membrane hyperpolarisation in six DLG TC neurons tested, which was augmented in the presence of OXA (control: −5.17 ± 0.7 mV; in OXA: 10.04 ± 1.6 mV, *p* = 0.0079, paired *t* test, n = 6; Fig. 5Ba–c). Interestingly, in six neurons out of 14 to which OXA was applied tonically, after adjusting the membrane potential to values preceding peptide application, irregular membrane oscillations/bursting were noted (Fig. 5Ba). The same bursting pattern was evoked by glutamate in the presence of OXA only in four out of six neurons that generated action potentials during their glutamate-evoked OXA-modulated responses (Fig. 5Ab).

### Calcium-dependent ionic mechanism underlies the effect of OXA in the DLG

**Figure 6:**
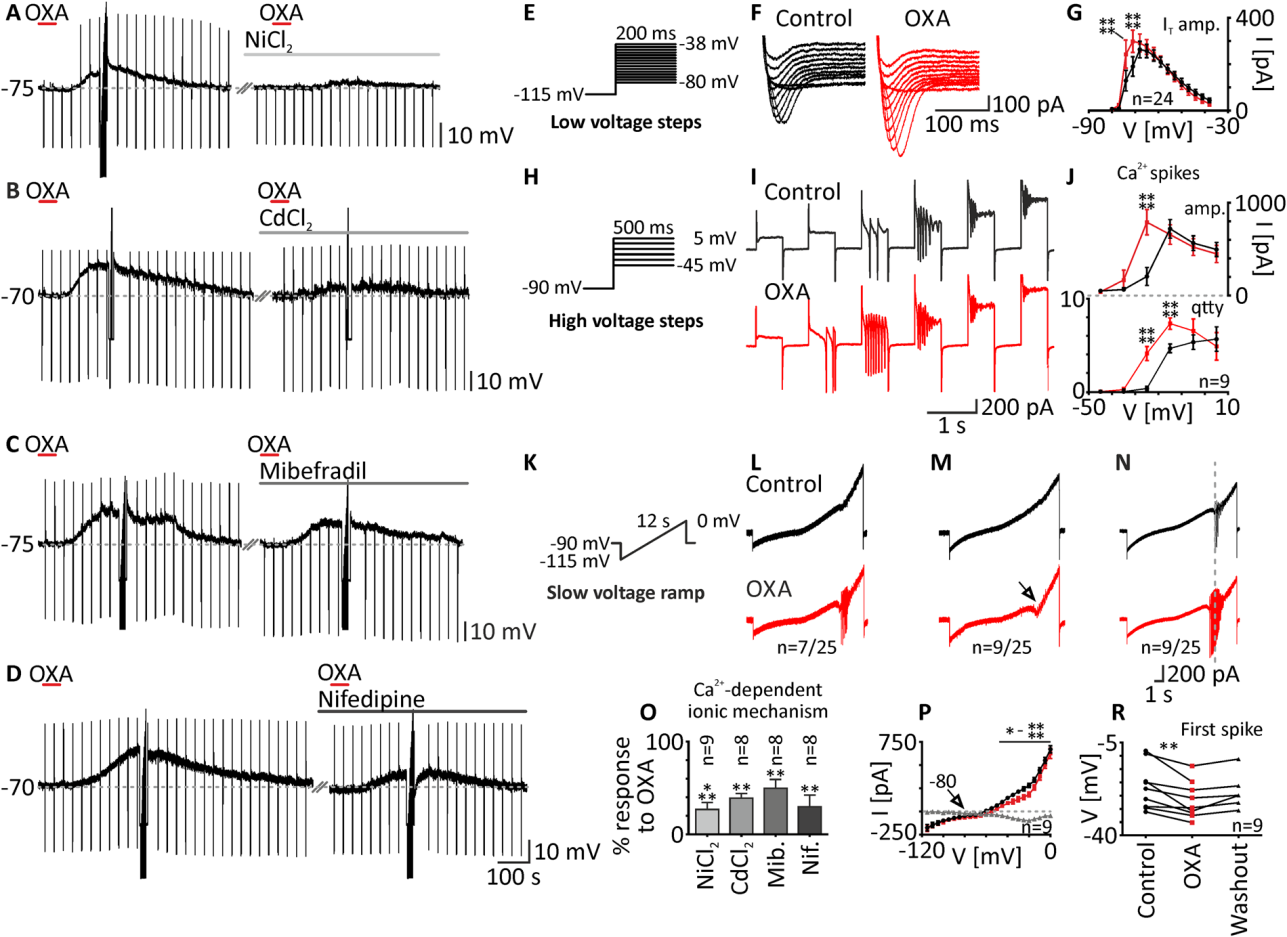
In DLG TC neurons, OXA evokes calcium influx through voltage-gated calcium channels. (**A**,**B**) Application of OXA (200 nM, red bars) produced a depolarisation that was heavily reduced by NiCl_2_ (10 mM, light grey bar) and CdCl_2_ (1 mM, grey bar). (**C**) Mibefradil (Mib, 30 μM, dark grey bar), reduced OXA-evoked depolarisation but in a less effective manner. (**D**) Nifedipine (Nif, 1 mM, black bar) also caused a reduction in the OXA effect. Responses to OXA (in%) in the presence of the blockers mentioned above are summarised as a bar graph (*O*). (**E**,**H**,**K**) Electrophysiological tests to elucidate calcium conductances were applied under control conditions (in TTX) and during the maximal response to OXA (200 nM). (**E**) Low voltage steps were used to evoke T-type calcium currents (I_T_). (**F**,**G**) Application of OXA enhanced the amplitude of I_T_ (in red). (**H**) In order to evoke HVA calcium currents, high voltage steps were used. (**I**) OXA caused the potentiation of HVA calcium spikes and a shift in their voltage dependency (in red). (**J**) Application of OXA resulted in an increase in calcium spike amplitude (amp.) and quantity (qtty). (**K**) Slow voltage ramps were also performed. Current responses under control conditions were further collated with those during responses to OXA (in red). (**L**) The first group of neurons examined (n = 7/25) was characterised by calcium spikes evoked by OXA application. (**M**) The second group tested (n = 9/25) lacked calcium spikes but elicited an inward current as a result of OXA treatment with a reversal potential of −80 mV and a significant augmentation in the voltage range between −45 mV and 0 mV (*P*). (**N**) The last group of neurons tested with slow voltage ramps (n = 9/25) showed calcium spikes under both control conditions and during the OXA effect. (**R**) The potential at which the first calcium spike was generated was shifted during OXA-evoked depolarisations. *p < 0.05, **p < 0.01, ***p < 0.001, ****p < 0.0001 denotes significance (Sidak’s multiple comparison test or paired *t* test). In the bar graphs, data are expressed as mean ± SEM. The number of examined neurons is shown as “n”. Downward deflections represent responses to current pulses (1 s, 80 pA).

In view of the considerable literature demonstrating the involvement of calcium channels in the excitatory effects of OXA (200 nM), we decided to check if calcium currents underlie OXA-evoked depolarisations of DLG TC neurons. All of the experiments concerning the ionic mechanism of OXA action were performed in the presence of TTX (0.5 μM). First, we tested the non-selective voltage-dependent calcium channels blocker - NiCl_2_ (10 mM) to nine DLG TC neurons. The addition of this agent to ACSF reduced the response to OXA to 26.78 ± 7.5% (control: 7.13 ± 1.0 mV; NiCl_2_: 1.63 ± 0.4 mV, *p* = 0.0002, paired *t* test, n = 9; Fig. 6A,O). Then, we tested CdCl_2_ (1 mM), which is more selective towards blocking the HVA calcium current. ACSF enriched with this blocker reduced the OXA-evoked depolarisation to 38.88 ± 5.0% in eight neurons recorded (control: 8.58 ± 1.6 mV; CdCl_2_: 3.18 ± 0.7 mV, *p* = 0.0021, paired *t* test, n = 8; Fig. 6B,O). In the next step, we decided to use specific blockers of distinct calcium conductances. To test if OXA activates LVA calcium currents, we applied this peptide in the presence of mibefradil (30 μM) to eight neurons. This blockage of the T-type calcium current resulted in a diminution in OXA-evoked depolarisation to 49.50 ± 9.6% (control: 10.01 ± 1.3 mV; Mib: 4.57 ± 1.1 mV, *p* = 0.0085, paired *t* test, n = 8; Fig. 6C,O). Similarly, we used nifedipine (1 μM) to block the L-type calcium current, a major contributor to the HVA calcium current in TC neurons. The addition of nifedipine to ACSF reduced the effect of OXA to 29.63 ± 12.6% in eight neurons examined (control: 8.69 ± 1.4 mV; Nif: 2.12 ± 0.9 mV, *p* = 0.0059, paired *t* test, n = 8; Fig. 6D,O). Low threshold spikes (LTS), elicited as a rebound depolarisation after recovery from current pulse-evoked hyperpolarisation, were diminished during the recordings in the presence of NiCl_2_ and mibefradil, but not CdCl_2_ and nifedipine.

To complement our pharmacological studies, we performed electrophysiological tests in the presence of TTX, elucidating possible modulation of different calcium currents by OXA (200 nM). First, low voltage steps (Fig. 6E) were used to uncover the T-type calcium current. Currents evoked by this test were sensitive to NiCl_2_ and mibefradil (data not shown). In 24 DLG TC neurons tested, OXA caused a significant augmentation in the T-type calcium current (*p* < 0.0001, two-way ANOVA, n = 24; Fig. 6F), in particular at the −74 mV (control: 129.23 ± 44.2 pA; in OXA: 242.16 ± 59.3 pA, *p* < 0.0001, Sidak’s test, n = 24; Fig. 6G) and −71 mV voltage steps (control: 190.21 ± 41.9 pA; in OXA: 298.99 ± 48.0 pA, *p* < 0.0001, Sidak’s test, n = 24; Fig. 6G). Second, in nine responsive DLG TC neurons, we used high voltage steps (Fig. 6H) to elicit the HVA calcium current manifested as calcium spikes, blocked by the application of CdCl_2_ and nifedipine (data not shown). Application of OXA changed the examined parameters of calcium spikes: it significantly enlarged their amplitude (*p* < 0.0001, two-way ANOVA, n = 9; Fig. 6I), particularly at the −25 mV voltage step (control: 205.59 ± 96.6 pA; in OXA: 790.65 ± 136.6 pA, *p* < 0.0001, Sidak’s test, n = 9; Fig. 6J), and increased their quantity (*p* < 0.0001, two-way ANOVA, n = 9) significantly at the −25 mV (control: 0.33 ± 0.2; in OXA: 4.11 ± 0.8, *p* < 0.0001, Sidak’s test, n = 9; Fig. 6J) and −15 mV voltage steps (control: 4.67 ± 0.47; in OXA: 7.33 ± 0.6, *p* < 0.0001, Sidak’s test, n = 9; Fig. 6J). Finally, we performed slow voltage ramps (Fig. 6K) on 25 DLG TC neurons. Based on the protocol used for the application of OXA and the type of current response to voltage, recorded neurons were divided into three different groups. The first one, represented by seven neurons (n = 7/25), was characterised by a lack of calcium spikes under control conditions; however, they were evoked during the response to OXA (Fig. 6L). The second group of neurons (n = 9/25) did not express calcium spikes under both conditions (Fig. 6M). In these neurons, the current response to the slow voltage ramp was sampled at 2 Hz (one data point every 5 mV) and further analysed. OXA was found to elicit an inward current that started at −80 mV (reversal potential) and was further augmented in the voltage range between −45 mV and 0 mV (*p* < 0.001, two-way ANOVA, n = 9; *p* = 0.0231 to *p* < 0.0001, Sidak’s test, n = 9; Fig. 6P), with the peak at −20 mV (difference current: −97.22 ± 20.3 pA, n = 9). The third, equally large group of neurons (n = 9/25), was characterised by the appearance of calcium spikes under both control conditions and after the application of OXA (Fig. 6N). In this group, we examined possible changes in the threshold of calcium spike generation evoked by OXA application. Indeed, OXA shifted the first spike potential towards more hyperpolarised values (control: −21.71 ± 2.8 mV; in OXA: −26.88 ± 2.3 mV, *p* = 0.0037, paired *t* test, n = 9; washout: −23.43 ± 2.8 mV, n = 6; Fig. 6R).

### Potassium current blockage by OXA is a supplementary ionic mechanism in DLG

**Figure 7:**
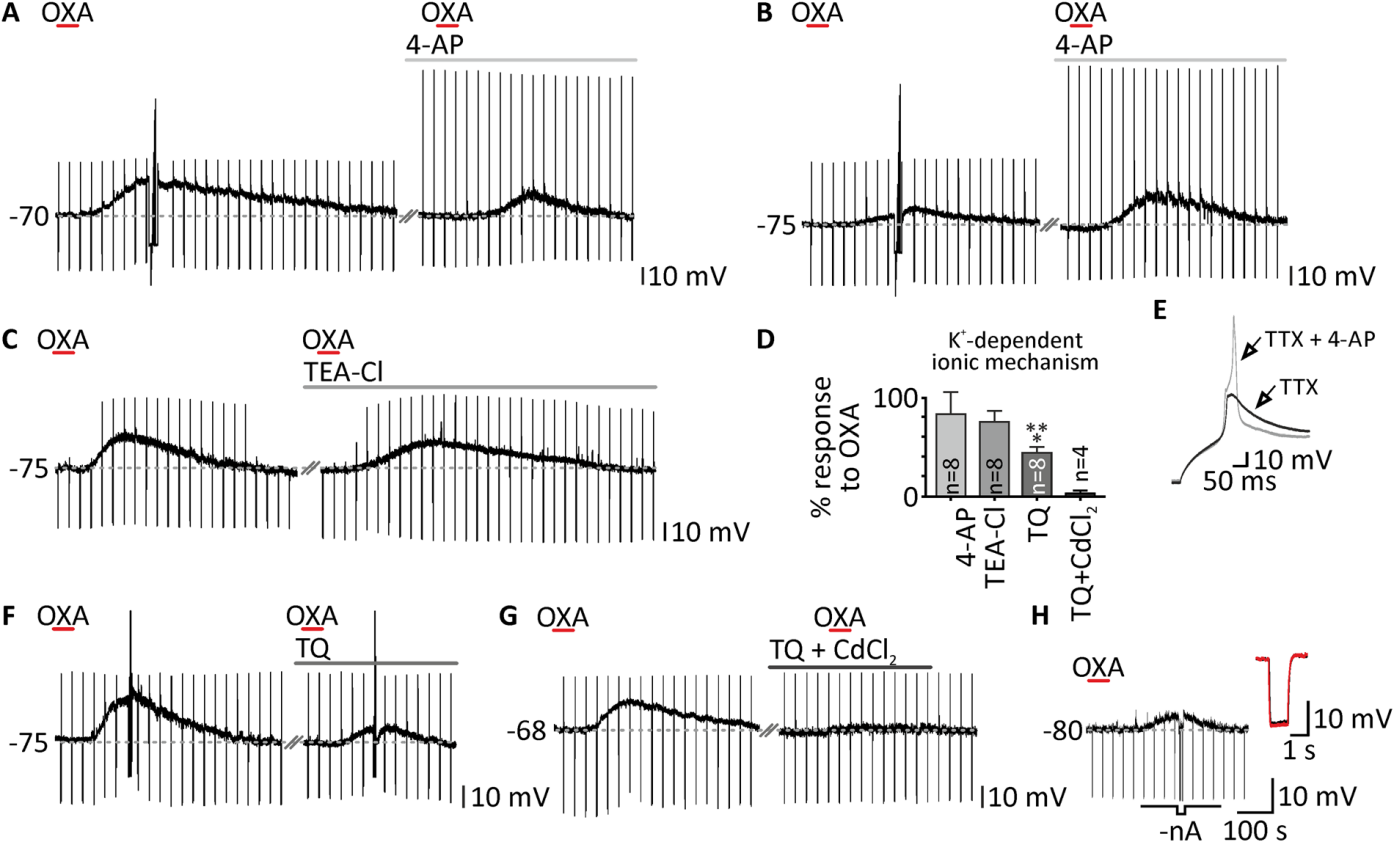
Selective closure of potassium conductance underlies the complementary ionic mechanism of OXA action in the DLG. (**A**,**B**) Response to OXA (200 nM, red bars) in the presence of a transient potassium current blocker 4-aminopirydine (4-AP, 1 mM, light grey bars) did not significantly differ from control conditions. Extreme examples of reduction and enhancement of OXA-evoked depolarisations by 4-AP treatment are shown in (**A**) and (**B**), respectively. The presence of 4-AP in the ACSF disinhibited the HVA calcium current and changed the shape of the rebound depolarisation after hyperpolarising current injection, as presented in (**E**). (**C**) Treatment with tetraethylammonium chloride (TEA-Cl, 10 mM, grey bar), a sustained potassium current blocker, was also unable to change the effects of OXA. (**F**) In the presence of tertiapin Q (TQ, 100 nM, dark grey bar), a GIRK channel blocker, depolarisations evoked by OXA were significantly reduced. (**G**) A cocktail of TQ and CdCl_2_ (100 nM and 0.5 mM, respectively, black bar) was able to entirely block OXA-evoked depolarisations. Responses to OXA (in%) left in the presence of the blockers mentioned above are summarised in a bar graph (**D**). (**H**) The membrane resistance was not changed by OXA application as shown by the comparison of voltage responses to current pulse in the baseline (in black) and during the plateau of response (in red) when potential was manually adjusted to control values (-nA). ***p < 0.0001 denotes significance (paired *t* test). In the bar graphs, data are expressed as mean ± SEM. The number of examined neurons is shown as “n”. Downward deflections in the raw current clamp traces represent responses to rectangular current pulses (1 s, 80 pA).

Since OXA-evoked depolarisation in DLG TC neurons did not entirely vanish in the presence of calcium current blockers, we subsequently turned to the possible inhibition of potassium conductances by OXA (200 nM). Furthermore, the action of OXA probably did not rely on opening of ionic channels alone, as we found that membrane resistance did not change significantly during the peptide-evoked depolarisation in seven neurons, although tested only in the native membrane potential (resistance in control: 438.14 ± 40.4 MΩ; in OXA, after adjusting membrane potential to control values: 449.00 ± 42.4 MΩ, *p* = 0.1084, paired *t* test, n = 7; Fig. 7H). First, we examined the possible involvement of the transient voltage-dependent potassium current. In the presence of 4-AP (1 mM), an A-type potassium current blocker, the effect of OXA was unchanged in the group of eight neurons tested (control: 7.78 ± 1.5 mV; 4-AP: 6.50 ± 1.6 mV, *p* = 0.4448, paired *t* test, n = 8; Fig. 7A,B,D). Similar results were obtained from a group of eight neurons where we applied OXA in the presence of TEA-Cl (10 mM; a sustained potassium current blocker). Again, the administered blocker did not significantly change OXA-evoked depolarisations (control: 10.42 ± 2.0 mV; TEA-Cl: 8.47 ± 2.2 mV, *p* = 0.1149, paired *t* test, n = 8; Fig. 7C,D). Interestingly, in the case of three neurons examined, the presence of voltage-dependent potassium channel blockers mildly augmented the response to OXA (presented at the Fig. 7B, as the strongest increase of OXA-evoked response in 4-AP). The application of 4-AP caused the disinhibition of HVA calcium currents, which resulted in rebound calcium spikes of a large amplitude (Fig. 7E). Then, we shifted our attention to G protein-coupled inwardly-rectifying potassium channels (GIRK), likely to be closed as an effect of orexin action. The application of tertiapin Q (100 nM) to eight DLG TC neurons significantly reduced the response to OXA to 44.38 ± 6.1% (control: 16.27 ± 2.0 mV; TQ: 7.61 ± 1.5 mV, *p* = 0.0004, paired *t* test, n = 8; Fig. 7F,D). Finally, we applied OXA in the presence of two blockers, i.e. tertiapin Q (100 nM) and CdCl_2_ (0.5 mM), to check whether the simultaneous blockage of GIRK and HVA calcium channels was sufficient to remove OXA-evoked depolarisation. Indeed, in four DLG TC neurons examined, OXA did not evoke any effect in the presence of tertiapin Q and CdCl_2_ together (control: 13.78 ± 5.4 mV; TQ + CdCl_2_: 0.39 ± 0.3 mV, n = 4; Fig. 7G,D).

### TC neurons localised in the anterior DLG are more likely to be depolarised by OXA

**Figure 8:**
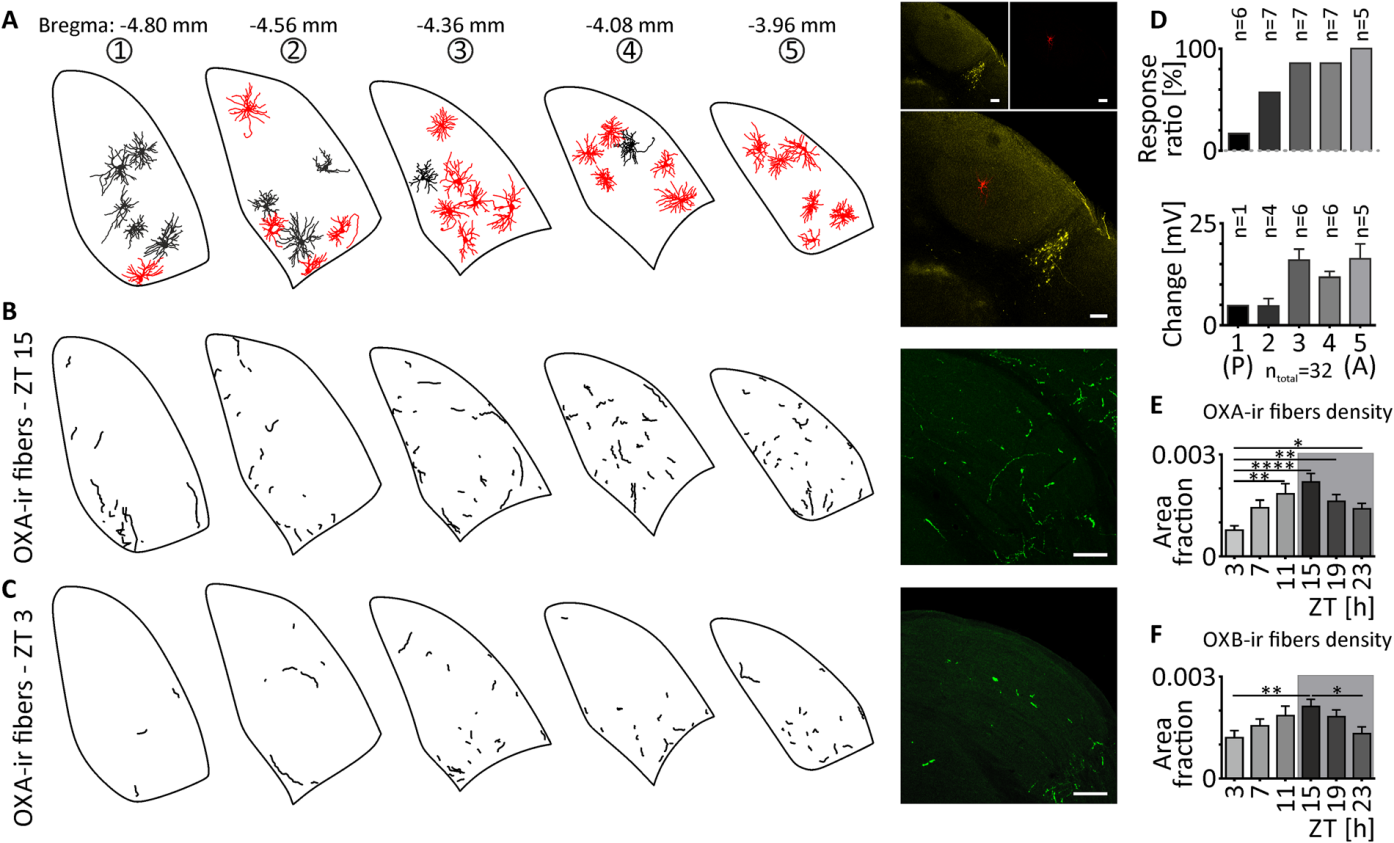
Anatomical representation of the influence of the orexinergic system in the DLG. (**A**) DLG TC neurons tested with OXA (200 nM) were filled with biocytin during the recording and drawn into a rat brain atlas^78^. Outlines of neurons that were depolarised by OXA are presented in red, in contrast to black ones that were found to be insensitive to the treatment. Note that sensitivity to OXA increases in the anterior DLG slices. Overlay of the exemplary confocal microphotography shows the localisation of recorded cell (in red) above the NPY-positive staining (in yellow). (**D**) Both the response ratio and depolarisation amplitude tend to increase in the anterior direction. (**B**–**F**) The density of orexinergic fibres in the DLG is sparse, but shows circadian rhythmicity. (**B**) OXA-immunoreactive (OXA-ir) fibres of the highest density were observed at 15 ZT (dark phase). (**C**) OXA-ir fibres at 3 ZT (light phase) were characterised by the lowest density. Exemplary staining is shown in green. (**E**) The circadian rhythm of OXA-ir fibres in the DLG was concluded from fibre density measurements at six time points (every four hours). (**F**) The density of OXB-immunoreactive (OXB-ir) fibres in the DLG shows similar circadian variability. Grey boxes indicate the dark phase. *p < 0.05, **p < 0.01, ***p < 0.001, ****p < 0.0001 denotes significance (Dunn’s multiple comparison test). In the bar graphs, data are expressed as mean ± SEM. “A” and “P” in the parentheses indicate anterior and posterior, respectively. White bars indicate 200 μm.

During our research, we noticed that the application of orexins to TC neurons localised in the anterior part of DLG was more likely to evoke a response. To check this hypothesis in a systematic way, we conducted separate set of experiments on seven hemispheres derived from four rats. Each DLG was cut into five coronal slices along the anterior-posterior axis. After electrophysiological recordings, slices were immunostained and drawn into the five closest sections of rat brain atlas (Fig. 8A). The results support our hypothesis, as OXA-evoked depolarisations were less frequent in the first posterior slice (slice no. 1; n = 1/6; Fig. 8D) and the most widespread in the most anterior DLG slice (no. 5; n = 5/5; Fig. 8D). Moreover, the increase in the frequency of responses was gradual along the anterior-posterior axis (slice no. 2: n = 4/7; slices no. 3 and no. 4: n = 6/7, each; Fig. 8D). The mean amplitude of responses to OXA (12.33 ± 1.2 mV, n = 22) was very similar to what was obtained in previous protocols. However, neurons localised in the anterior part of the DLG were characterised by larger depolarisations in comparison to the posterior area (slice no. 1: 4.79 mV, n = 1; no. 2: 4.69 ± 1.9 mV, n = 4; no. 3: 15.97 ± 2.7 mV, n = 6; no. 4: 11.76 ± 1.4 mV, n = 6; no. 5: 16.28 ± 3.7 mV, n = 5; Fig. 8D).

### Orexinergic innervation of the DLG is sparse but shows a circadian rhythm

Finally, we performed a set of immunohistochemical experiments to address the question regarding sparse orexinergic innervation of the DLG and its possible diversity along circadian time. The quantification of OXA-ir fibres at the area of the DLG revealed a clear circadian rhythm (*p* < 0.0001, Kruskal^−^Wallis test; Fig. 8E) with the highest density of fibres at 15 ZT (area fraction: 0.00219 ± 0.0002; Fig. 8B,E) and a nadir at 3 ZT (area fraction: 0.00076 ± 0.0001; Fig. 8C,E). Next, we examined the rhythm of OXB in the DLG, and similar results were obtained (*p* = 0.0011, Kruskal Wallis test; Fig. 8F). The density of OXB-ir fibres was lowest at 3 ZT (area fraction: 0.00119 ± 0.0008; Fig. 8F) and highest at 15 ZT (area fraction: 0.00211 ± 0.0002; Fig. 8F).

### Orexins activate DLG TC neurons during the dark phase via the same ionic mechanism

As an additional set of experiment, we performed patch-clamp recordings on the DLG neurons derived from five rats sacrificed in darkness at 15 ZT (the highest density of OXA/OXB-immunoreactive fibres). All of the recordings were performed in the presence of TTX (0.5 µM). We applied OXA (200 nM) on 13, and OXB (200 nM) on 11 DLG TC neurons. Ten of the recorded cells were depolarised by OXA (control: −66.52 ± 1.5 mV; OXA: −61.44 ± 1.7 mV, *p* = 0.0001, paired *t* test, n = 10; Supplementary Fig. S1) and eight by OXB (control: −63.41 ± 1.4 mV; OXB: −56.36 ± 2.1 mV, *p* = 0.0013, paired *t* test, n = 8; Supplementary Fig. S1). The amplitudes of evoked depolarisations did not vary significantly between 1–2 ZT and 15 ZT (OXA: 9.07 ± 1.5 mV, n = 18 vs. 5.09 ± 0.8 mV, n = 10, *p* = 0.0774, unpaired *t* test; OXB: 7.97 ± 1.5 mV, n = 8 vs. 7.04 ± 1.4 mV, n = 8, *p* = 0.6496, unpaired *t* test; 1–2 and 15 ZT, respectively; Supplementary Fig. S1). Next, we moved to the verification of ionic mechanism underlying OXA-evoked depolarisations during the dark phase. The presence of tertiapin-Q (100 nM) and CdCl_2_ (0.5 mM) in the ACSF potently blocked the OXA-evoked depolarisations in four neurons tested (control: 7.03 ± 0.8 mV; TQ + CdCl_2_: 0.46 ± 0.2 mV; 5.96 ± 1.8% of response left, n = 4; Supplementary Fig. S1), what resembles the experiments in the light phase. Additionally, slow voltage ramps were used in eight OXA-responsive DLG TC neurons to electrophysiologically dissect the ionic currents responsible for observed depolarisations. The inward currents evoked by OXA at 15 ZT were very similar to those observed at 1–2 ZT (Supplementary Fig. S1).

## Discussion

In the present study, we demonstrated that orexins directly activate DLG TC neurons, predominately via postsynaptic OX_2_ receptors. Moreover, OXA increases neuronal excitability and sensitivity to both glutamate and GABA. Mechanistic studies suggest the involvement of both voltage-dependent calcium influx and closure of GIRK channels in OXA-evoked depolarisation. Immunohistochemical analysis revealed a circadian rhythm in the density of orexinergic fibres in the area of the DLG.

The data presented in this article clearly indicate that orexins are powerful excitatory modulators of neuronal activity in the DLG. This is supported by the fact that OXA depolarises the majority of DLG TC neurons in normal ASCF, in TTX, and in the presence of fast synaptic transmission blockers (CNQX, AP-5 and bicuculline). Our experiment concerning the possible involvement of both orexin receptors showed that, despite the action of OXA on the OX_1_ receptor, the OX_2_ receptor appears to be predominately active in the DLG on the postsynaptic side. In previous studies, only the OX_1_ receptor had been reported^46^. The role of both orexin receptors is therefore distinct for different parts of the lateral geniculate complex: the DLG and VLG are characterised by predominant activity of the OX_2_ receptor^43^, whereas in the IGL, OX_1_ and OX_2_ receptors are thought to contribute equally to OXA-evoked effects^42^.

Intriguingly, previous reports by Bayer *et al*. (2002) and Govindaiah and Cox (2006) showed that DLG neurons recorded by both groups were insensitive to OXA or OXB treatment. However, those studies do not focus on the DLG itself. Instead, they provided a comprehensive overview of thalamic sensitivity to orexins. In order to examine possible differences between our study and those of others, we have shown that depolarising effects of orexins are rat strain-independent and persist with matched anaesthesia and intrapipette chloride concentrations^33^. Interesting observations were provided by systematic mapping of responsive DLG TC neurons along the anterior-posterior axis. It turned out that the anterior parts of the DLG are much more sensitive to OXA application, in terms of both effect frequency and amplitude. Thus, our hypothesis regarding the observed discrepancies between our studies and previous publications is based on two assumptions. First, in previous research, the authors may have examined posterior parts of the DLG, where OXA-evoked depolarisations are very rare, and used the anterior slices for studies on different thalamic nuclei. Second, the number of observations is highly variable between our study (orexins were applied to 293 DLG TC neurons in the presence of TTX) and in previous reports in which the total number of DLG neurons tested was 6^12^ or 21^33^.

One of the principal findings of this study, which may shed the light on the possible functional implications of OXA in the DLG, is the sustained increase in TC neuron excitability accompanying OXA-evoked depolarisation. This phenomenon, that could possibly stem from membrane resistance changes at various membrane potentials, has been previously observed in other thalamic nuclei^33^. In our study, this was observed as an increased number of action potentials fired during the same current stimulation in the presence of OXA and a lowered threshold for sodium spike generation. Moreover, the results presented in this article show that tonic application of OXA can enhance responses to both excitatory glutamate and inhibitory GABA. This augmentation of response can be due to enhanced membrane trafficking of glutamate/GABA receptors or their direct modulation via intracellular pathways activated by OXA^47, 48–50^. Also, orexin receptors and metabotropic glutamate receptors/GABA_B_ receptors share common effectors eg. GIRK channels, what can underlie the potentiation of gltamate/GABA-evoked responses in the presence of OXA^51^. It is generally thought that orexins, similar to other neurotransmitters of arousal, play a role in maintaining wakefulness and facilitating cortical activation^20, 21, 34, 35, 52–57^. Here, we propose that these peptides, secreted under certain behavioural conditions, may facilitate information collection and flow through the thalamo-cortical pathway of the primary visual system.

As observed in previous studies, the opening of voltage-gated calcium channels constitutes a common target of orexin signalling. Both LVA and HVA calcium channels^9, 10, 36, 58–60^ were found to be modulated by OXA treatment. Therefore, we tested the possibility that calcium influx may be a mechanism underlying the OXA-mediated depolarisation of DLG TC neurons. Collectively, our experiments using calcium channel blockers and electrophysiological tests indicate that OXA enhances voltage-dependent calcium currents in the DLG, which may partially underlie its excitatory effect. These results are consistent with the fact that tonic administration of OXA and the application of glutamate in the presence of OXA induced sustained bursting, not present under control conditions. It is known that calcium influx, and therefore a reduction in the calcium concentration in the extracellular space, potentiates bursting^61, 62^.

Nevertheless, the absence of changes in membrane resistance (in the native membrane potential), residual depolarisation in the presence of calcium channel blockers and increased excitability led us to the idea that closure of potassium channels may additionally underlie OXA-evoked depolarisations. The involvement of potassium channels in the OXA effect has been previously noted in other brain structures^12, 15, 16^. More precisely, constitutively active GIRK channels have been shown to be closed by OXA, resulting in depolarisation in different brain structures^13, 14^. Indeed, in the case of DLG TC neurons, closure of GIRK channels was confirmed to underlie a part of OXA-evoked depolarisation. Most importantly, the results presented in this article indicate that combined blockade of GIRK and voltage-gated calcium channels was sufficient to completely inhibit OXA-evoked depolarisation. This observations were strengthened by the results of electrophysiological tests (slow voltage ramps) – the net orexin-induced current was characterised by the reversal potential of −80 mV (close to reversal potential of potassium) and a major rise in the voltage range from −45 mV to 0 mV (activation of voltage-dependent calcium conductance). Other, alternative mechanism of orexin action that reverses around −25 mV would be the activation of non-selective cationic conductance^63^. The closure of potassium channels is a common mechanism shared by many arousal-promoting neuromodulators derived from the brain stem, and has been found to alter the activity of thalamic nuclei^64–67^. This similarity may provide insight into the possible functional significance of OXA in the DLG.

At present, the source of orexins in the DLG remains unclear. Previous immunohistochemical studies indicated no or very sparse innervation of this nucleus^4, 68, 69^. Our new results show that, during the active phase (night in rodents), the density of OXA-ir fibres was nearly tripled, similarly for OXB-ir terminals (nearly doubled), in comparison with the beginning of the light phase. This result is consistent with the circadian rhythmicity of orexin synthesis in neurons of the lateral hypothalamus, as well as their activation in darkness^23, 24^. Observed changes in immunoreactivity can stem from both clock-dependent circadian changes and temporal pattern of levels of behavioural activities (reflecting the behavioural arousal levels during day and night). Still, even at the time of the highest density of orexin-ir fibres, it seems unlikely that this limited number of terminals would be sufficient to synaptically contact as many responsive TC neurons. However, this kind of transmitter-receptor mismatch is not unusual in the brain, as many neuromodulators that do not have to act in short temporal windows use volume transmission^70^. This is not uncommon for neuropeptides that act in nanomolar concentrations, possess a long extracellular half-life, and penetrate the tissue for long distances^71, 72–74^. The close proximity of the IGL, densely innervated by orexinergic cells, as well as the lateral ventricle, could favour this type of transmission^4, 69^. Based on the presented rationale, we hypothesise that volume transmission could be responsible for the presence of orexins in the DLG.

Interestingly, the additional experiments performed during the dark phase (15 ZT), when the density of orexin-immunoreactive fibres is the highest, shown no significant differences in response amplitude, frequency and ionic mechanism, in comparison to light phase. The slight decrease in the amplitude of OXA-evoked depolarisations may stem from the possible receptor down-regulation by the higher concentration of agonist at night. Altogether, our findings indicate that DLG neurons are able to respond to orexins very similarly during the day and night. Therefore, the modulatory effects of orexins in the DLG lies with the activity of orexinergic cells, dependent both on the circadian time and arousal^24^.

A clear connection between discharge strength, the pattern of thalamic neurons, and the state of arousal has been established^25, 75, 76^. Moreover, circadian modulation of visual sensitivity and light-induced responses in the DLG, dependent on the suprachiasmatic nuclei, have been described^32, 77^. As its best-known function, the orexinergic system is sensitive to arousal and mediates its effects by activating various thalamic and cortical areas. Also, its activity depends heavily on circadian time. Therefore, due to extensive connections, orexinergic neurons can modulate the activity of many brain circuits in a circadian time-dependent manner^24, 40^. Here, we show that orexins are powerful excitatory modulators of DLG TC neurons and show circadian rhythmicity in the studied structure. In the light of the presented data, the orexinergic system is a good candidate for mediating circadian and arousal-related activation to modulate visual processes at the level of the DLG.

## Materials and Methods

### Animals

All experiments were approved by the Local (Krakow) Ethical Commission and performed in accordance with the European Community Council Directive of 24 November 1986 (86/0609/ EEC) and the Polish Animal Welfare Act of 23 May 2012 (82/2012). Every effort was made to minimise the number of animals used and their suffering. Animals were maintained in the Jagiellonian University animal facility on a standard 12 h light/dark cycle (lights on at 8:00 am, lights off at 8:00 pm) with water and food *ad libitum*. Constant environmental conditions (temperature of 23 °C, ~60% humidity) were provided.

### Tissue preparation

Electrophysiological experiments were performed on thalamic slices obtained from 2- to 3-week old male rats. Wistar rats were used for the majority of recordings, although in order to check the strain-specificity of the observed orexin effects, albino Sprague Dawley and pigmented Long Evans rats were also tested. Rats were anaesthetised with isoflurane (2 ml/kg body weight; Baxter, Deerfield, USA) before decapitation between 9:00 and 10:00 a.m. (Zeitgaber Time: ZT 1–2), unless stated otherwise. One experimental group of Sprague Dawley rats was anaesthetised with sodium pentobarbital (100 mg/kg body weight, i.p.; Biowet, Pulawy, Poland) to determine whether the choice of an anaesthetic agent influenced subsequent responses to orexin application. Tissue preparation was previously described in detail^42^. Briefly, the brain was removed from the skull and immediately placed in ice-cold artificial cerebro-spinal fluid (ACSF) composed of (in mM): 118 NaCl, 25 NaHCO_3_, 3 KCl, 1.2 NaH_2_PO_4_, 2 CaCl_2_, 2 MgCl_2_ and 10 glucose (osmolality ~290 mOsmol/kg), gassed with 95% O_2_ and 5% CO_2_. Block of tissue containing the thalamus was placed on the frozen plate of a vibroslicer (Leica VT1000S, Heidelberg, Germany) and cut into 250 µm-thick coronal slices. Those containing the lateral geniculate complex were transferred to the pre-incubation chamber (initial temperature: 32 °C) for at least one hour (while cooling down to 21–24 °C), then placed in the recording chamber (21–24 °C).

### Electrophysiology

Borosilicate glass pipettes (Sutter Instruments, Novato, USA; resistance 5–9 MΩ) were placed above the DLG under visual microscopic control. Next, single DLG neurons were identified with a Zeiss Examiner microscope fitted with infrared differential interference contrast (Göttingen, Germany) under a 40x magnifying objective. An Ez-gSEAL100B Pressure Controller (Neo Biosystem, San Jose, USA) was then used to approach the neuron and obtain the whole-cell configuration. The recorded signal was amplified by a SC 05LX (NPI, Tamm, Germany) amplifier, low-pass filtered at 2 kHz and digitised at 20 kHz. Spike2 and Signal (Cambridge Electronic Design Inc., Cambridge, UK) software were used for all recordings. A liquid junction potential of −14.8 mV was added to the measured values of membrane potential. Patch pipettes were filled with an intrapipette solution containing (in mM): 125 potassium gluconate, 20 KCl, 10 HEPES, 2 MgCl_2_, 4 Na_2_ATP, 0.4 Na_3_GTP, 1 EGTA and 0.05% biocytin (pH 7.4 adjusted with 5 M KOH; osmolality ~300 mOsmol/kg). Under those conditions, the reversal potential for chloride anions (R_Cl_^−^) amounted to about −43 mV. In order to exclude the possibility that a high Cl^−^ concentration in the intrapipette solution may cause discrepancies between our results and others^12, 32^, low Cl^−^ intrapipette solution (R_Cl_^−^ = −70 mV) was prepared, containing (in mM): 138 potassium gluconate, 3 KCl, 10 HEPES, 2 MgCl_2_, 4 Na_2_ATP, 0.4 Na_3_GTP, 1 EGTA and 0.05% biocytin (pH 7.4 adjusted with 5 M KOH; osmolality ~300 mOsmol/kg).

All experiments were conducted in current clamp mode (holding current = 0 nA) at room temperature (21–24 °C). In order to monitor membrane resistance, rectangular current pulses (1 s, 80 pA) were adjusted every 30 seconds throughout the recording. Neurons that changed their membrane resistance by more than 15% during the recording were excluded from further analysis.

To elucidate low- and high-voltage activated calcium currents (LVA and HVA, respectively), tests were performed in voltage clamp mode. For LVA, depolarising steps (15 steps, 200 ms duration, 700 ms interval) were given in 3 mV increments (first step at −80 mV) from the holding potential of −115 mV (Fig. 6E). For HVA, similar steps (6 steps, 500 ms duration, 500 ms interval) were applied in 10 mV increments (first step at −45 mV) from the holding potential of −90 mV (Fig. 6H). Slow voltage ramps (12 s duration, from −115 mV to 0 mV, holding potential = −90 mV; Fig. 6K) were also performed. To study excitability changes, a trapezoidal current test was applied (5 s total duration: 0.6 s ramp from −150 pA to +150 pA followed by 4.4 s plateau at +150 pA; Fig. 6I,C).

### Immunohistochemical verification

After each experiment, slices containing recorded neurons were processed for staining in order to verify the location of recorded cells in the investigated structure and visualise their morphology. First, immediately after recording, slices were fixed in 4% paraformaldehyde (PFA) in phosphate-buffered saline (PBS) at 4 °C overnight. Subsequently, slices were placed in permabilising solution containing 0.6% Triton X-100 (Sigma-Aldrich, Schnelldorf, Germany) for three to four hours at room temperature. Finally, slices were incubated with Cy3-conjugated Extravidin (1:250, Sigma-Aldrich, Schnelldorf, Germany) in PBS containing 0.3% Triton-X100 at 4 °C overnight. In one set of electrophysiological experiments, in order to draw sections onto a rat brain atlas, slices were immunostained against neuropeptide Y (NPY) to visualise the shape of the intergeniculate leaflet to better define the anterior-posterior diameter. Consequently, 10% normal donkey serum (NDS; Jackson ImmunoResearch, West Grove, PA, USA) was added to the permabilising solution. Next, slices were incubated with rabbit NPY antisera (1:8000, Sigma-Aldrich, Schnelldorf, Germany) in PBS, 2% NDS, 0.3% Triton-X100 and Cy3-conjugated Extravidin (1:250, Sigma-Aldrich, Darmstad, Germany) at 4 °C overnight. Finally, slices were rinsed twice in PBS and incubated with anti-rabbit AlexaFluor 647-conjugated antisera (1:300; Jackson ImmunoResearch, West Grove, USA) at 4 °C overnight. At the end of the procedure, slices were rinsed twice in PBS and mounted on glass slides in Fluoroshield™ (Sigma-Aldrich, Schnelldorf, Germany). Brain slices were viewed under an A1-Si Nikon Inc. (Japan) confocal laser scanning system built on an inverted microscope Nikon Ti-E (Japan). Biocytin-filled neurons were classified as thalamo-cortical if they were characterised by large, round soma and three or more primary dendrites (multipolar cell), in contrast to interneurons, with a small, spindle-shape soma with two primary dendrites (bipolar cell; Fig. 1A).

### Reagents

OXA (100 or 200 nM; Tocris, Bristol, UK), OXB (200 nM; Sigma-Aldrich, Schnelldorf, Germany), tetrodotoxin citrate (TTX, 0.5 µM; Tocris, Bristol, UK), the selective OX_2_ receptor antagonist TCS OX2 29 (TCS, 10 µM; Tocris, Bristol, UK), 6-cyano-7-nitroquinoxaline-2,3-dione (CNQX, 10 µM; Tocris, Bristol, UK), 2-amino-5-phosphonopentanoic acid (AP-5, 40 µM; Tocris, Bristol, UK), bicuculline methiodide (Bic, 20 µM; Tocris, Bristol, UK), mibefradil dihydrochloride (Mib, 30 µM; Tocris, Bristol, UK), nifedipine (Nif, 1 µM; Tocris, Bristol, UK), 4-aminopirydine (4-AP, 1 mM; Tocris, Bristol, UK), tetraethylammonium chloride (TEA-Cl, 10 mM; Tocris, Bristol, UK) and tertiapin-Q (TQ, 100 nM; Tocris, Bristol, UK) were kept as stock solutions (100x concentrated, dissolved in 0.9% NaCl) at −20 °C and prepared fresh as working solution in ACSF prior to each experiment. γ-amino butyric acid (GABA, 100 µM; Sigma-Aldrich, Schnelldorf, Germany) and monosodium glutamate (Glu, 200 µM; Sigma-Aldrich, Schnelldorf, Germany) were stored as 100x concentrated stocks in 0.9% NaCl at 4 °C. Nickel chloride (NiCl_2_, 10 mM; Sigma-Aldrich, Schnelldorf, Germany) and cadmium chloride hydrate (CdCl_2_, 0.5 or 1 mM; Sigma-Aldrich, Schnelldorf, Germany) were weighed and dissolved in ACSF separately for each recording. SB 334867 (SB, 10 μM; Tocris, Bristol, UK), a selective non-peptide OX_1_ receptor antagonist, was dissolved in 100% dimethyl sulfoxide (DMSO; Sigma-Aldrich, Schnelldorf, Germany) and stored as a 1000x concentrated stock solution at −20 °C. Therefore, the maximal concentration of DMSO in ACSF during the recording was 0.1%. All drugs were delivered by bath perfusion and approximately 150 s was needed for the substance to reach the recording chamber via the tubing system.

### Data analysis and statistics

Electrophysiological recordings were analysed with the use of custom made scripts in Spike2 (Cambridge Electronic Design Inc., Cambridge, UK) and MATLAB (MathWorks, Inc., USA). Changes in the membrane potential and input resistance were considered to be significant if they differed by more than three standard deviations (SDs) from the averaged baseline values. All data are described as the highest point of the examined value reached during the response and expressed as the mean value ± SEM. Statistical analysis was performed in GraphPad Prism 6 (GraphPad Software, Inc. USA). The Shapiro-Wilk normality test, Student’s paired and unpaired *t* tests, Wilcoxon test, one-way ANOVA followed by Tukey’s or Kruskal-Wallis multiple comparisons tests, the Friedman test followed by Dunn’s multiple comparisons test and two-way ANOVA followed by Sidak’s multiple comparisons test were used and *p* < 0.05 was considered significant.

### Orexinergic fibres immunohistochemistry and image processing

Adult male Wistar rats (250 g–350 g; n = 24) were anesthetised in two steps: with isoflurane (2 ml/kg body weight; Baxter, Deerfield, USA) and sodium pentobarbital (100 mg/kg body weight, i.p.; Biowet, Pulawy, Poland). Then, rats were submitted to a tissue fixation process by paraformaldehyde (4%, in PBS) transcardial perfusion. Next, the brains was removed from the skull and post-fixed in the same fixative solution overnight. In order to study the circadian distribution of orexinergic fibres in the DLG, the described procedure was performed at 4 h intervals on groups of four rats each, over a 24-h period (starting at ZT 3). Due to unsuccessful fixation, one rat brain from 11 ZT was excluded from further analysis.

Tissue was cut on a vibroslicer (Leica VT1000S, Heidelberg, Germany) in 50 µm coronal sections containing the DLG. After sectioning, slices were transferred to permabilising solution containing 0.6% Triton-X100 (Sigma-Aldrich, Schnelldorf, Germany) and 10% normal donkey serum (NDS, Jackson ImmunoResearch, West Grove, PA, USA) in PBS at room temperature. After 35 min, slices were washed in PBS and incubated with rabbit NPY antisera (1:8000, Sigma-Aldrich, Schnelldorf, Germany) and goat OXA or OXB antisera (1:200, Santa Cruz Biotechnology, CA, USA) in PBS containing 2% NDS and 0.3% Triton-X100 overnight at 4 °C. In the following step, slices were washed twice in fresh PBS and incubated with anti-rabbit AlexaFluor 647 and anti-goat AlexaFluor 488 conjugated antisera (both 1:300; Jackson ImmunoResearch, West Grove, PA, USA) overnight at 4 °C. Finally, sections were placed on glass slides, dried and coverslipped with Fluoroshield^TM^ (Sigma-Aldrich, Schnelldorf, Germany).

Confocal images were collected on an A1-Si Nikon Inc. (Japan) confocal laser scanning system built on an inverted Nikon Ti-E microscope (Japan). The area containing the whole lateral geniculate complex was scanned. Image processing was performed with the use of ImageJ2 software (v. 2.0.0-rc41/1.50d, NIH, USA) for optimal representation of the data. Quantitative analysis of the orexinergic fibres was performed in three steps. At first, Bernsen’s adaptive thresholding method was used for raw digital images to define regions of high local contrast. Then, the area corresponding to the DLG was selected. Finally, the “Analyse Particles” command was used to count immunoreactive pixels corresponding to orexinergic fibres. All digital images were processed with the same settings. The final results are reported as the total sum of immunoreactive pixels divided by the area of the DLG (area fraction) ± SEM. Data were analysed in GraphPad Prism 6 (GraphPad Software, Inc. USA) with one-way ANOVA followed by the Kruskal-Wallis multiple comparisons test; *p* < 0.05 was considered significant.

## Electronic supplementary material

Supplementary Information
