# Supplementary material for: Multiple excitatory actions of orexins upon thalamo-cortical neurons in dorsal lateral geniculate nucleus - implications for vision modulation by arousal
Source: Sci Rep. 2017 Aug 9;7:7713. doi: 10.1038/s41598-017-08202-8 (PMC5550457; doi:10.1038/s41598-017-08202-8)
Supplement: Supplementary file 1 — Supplementary Information [file 41598_2017_8202_MOESM1_ESM.doc]

**Title:** Multiple excitatory actions of orexins upon thalamo-cortical neurons in dorsal lateral geniculate nucleus - implications for vision modulation by arousal

**Authors:** Chrobok Lukasz^1^, Palus Katarzyna^1^, Chrzanowska Anna^1^, Kepczynski Mariusz^2^, Lewandowski Marian Henryk^1*^

^1^Department of Neurophysiology and Chronobiology, Institute of Zoology and Biomedical Research, Jagiellonian University in Krakow, Gronostajowa 9 Street, 30-387 Krakow, Poland

^2^Faculty of Chemistry, Jagiellonian University in Krakow, Ingardena 3 Street, 30-060 Krakow, Poland


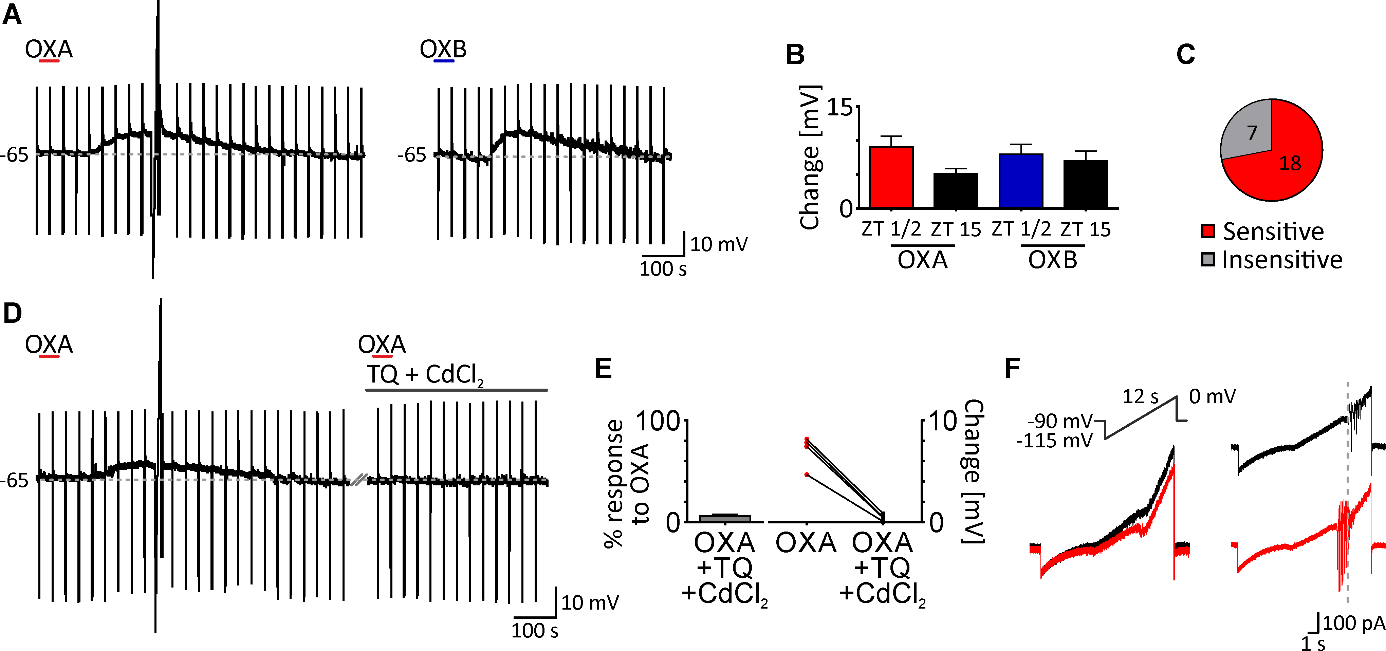


Supplementary Figure S1. Orexins activate DLG TC neurons during the dark phase via the same ionic mechanism. *A.* Example recordings showing OXA- (200 nM, red bar) and OXB-evoked (200 nM, blue bar) depolarisations during the night. *B*. The amplitude of the responses did not differ significantly between dark (ZT 15, black bars) and light phase (ZT 1/2, red and blue bars). *C.* The total proportion of orexin-responsive neurons was very similar to the one obtained during the day. *D,E.* Example recording and summary graphs showing the effects of tertiapin-Q (100 nM, TQ) and CdCl_2_ (0.5 mM) on the OXA-evoked depolarisations. *F.* Two examples of OXA action on slow voltage ramp-evoked currents. The inward currents evoked by OXA application at night did not differ to those observed during the day. In the bar graph, data are expressed as mean ± SEM. Downward deflections in the raw current clamp traces represent responses to rectangular current pulses (1 s, 80 pA).
